# Addressing methodological and interpretive issues in the study of high-energy focused ESWT for chronic lumbar facet syndrome

**DOI:** 10.1097/JS9.0000000000002794

**Published:** 2025-06-24

**Authors:** Xianwen Sun, Jiekun Jian

**Affiliations:** The Second School of Clinical Medicine, Zhejiang Chinese Medical University, Hangzhou, Zhejiang, China

*Dear Editor,*

We read with interest the study by Nedelka *et al* (2025) reporting that high-energy focused ESWT significantly improved pain (VAS) and function (ODI) in chronic lumbar facet syndrome, with 64% reduction in VAS at 12 months and imaging evidence of bone marrow edema resolution^[1]^. We commend the authors for investigating a non-invasive alternative for facet pain. However, we note several methodological and interpretive issues that warrant careful consideration. In line with the TITAN 2025 guideline on AI transparency^[2]^, a generative AI–based language model was used solely for language polishing and had no bearing on the scientific content.

First, key details of the trial design are lacking. The authors state that facet pain was “confirmed by medial branch block,” but do not specify whether single or dual comparative blocks were used. A single block can have high false-positive rates; standard practice often requires confirmatory blocks to increase diagnostic specificity. Similarly, the randomization and blinding procedures are not described in detail. No information is provided on allocation concealment, sham procedure blinding, or participant flow (dropout rates) over 12 months. Nor do the authors report a sample-size calculation, raising uncertainty about study power. These omissions are contrary to CONSORT reporting standards^[3]^, which recommend transparent disclosure of randomization methods, blinding, and attrition. Without these details, bias may undermine the validity of the findings.

Second, the statistical interpretation appears optimistic. The reported effect size (Cohen’s *d* = 1.12) is unusually large for a pain intervention, which is prone to placebo effects and regression to the mean. Multiple outcomes (VAS, ODI, PainDETECT at 2, 6, 12 months) were tested without correction for multiple comparisons; thus, some reported *P*-values may be false positives. As Nuzzo cautions, reliance on *P* < 0.05 without prespecified analysis plans can be misleading^[4]^. In particular, the very large pain reduction in the ESWT group (64.4% at 12 months vs 12.5% in sham) is unexpected. It suggests either an extraordinary treatment effect or potential unblinding/placebo influences. In light of the large number of statistical tests performed, we would like reassurance that the analyses were prespecified and adequately powered, and that any dropouts were handled appropriately.

Third, the mechanistic claims merit caution. The authors ascribe the improvements partly to “resolution of bone marrow edema” seen on MRI. However, the biologic basis by which ESWT would reverse Modic-type changes in facet joints is not established. The assertion that MRI changes confirm a specific therapeutic effect is speculative without further validation. Moreover, the improvement in neuropathic pain score (PainDETECT) is surprising, since facet joint pain is generally nociceptive in origin; it is unclear how ESWT would selectively reduce neuropathic symptoms. It would help to have a discussion of plausible mechanisms (e.g. anti-inflammatory or remodeling effects) and any supporting preclinical data. We also note that a high-quality randomized trial found that radiofrequency facet denervation produced no clinically important benefit over exercise^[5]^. That finding underscores how difficult it has been to demonstrate meaningful facet pain relief even with targeted interventions. The dramatic benefits reported here for ESWT—far exceeding those for radiofrequency—therefore appear to conflict with existing evidence. The authors should address how their results align with current understanding of facet joint pathology and treatment.

In conclusion, while Nedelka *et al* introduce an intriguing approach, the above concerns limit confidence in the conclusions. We suggest that future work include rigorous blinding, adequate power, and pre-registered analysis. In its present form, the study’s claims (especially concerning MRI changes and neuropathic pain) should be interpreted with caution.

## Data Availability

Not applicable.

## AI assistance

A generative AI-based language model was employed solely for grammar and style editing.
